# Stories of who we are: exploring trainee pharmacists’ professional identity constructions through workplace narratives

**DOI:** 10.1007/s10459-025-10480-1

**Published:** 2025-10-03

**Authors:** Victoria Ruth Tallentire, Scott McColgan-Smith, Fiona Stewart, Samantha Eve Smith

**Affiliations:** 1 NHS Lothian, Edinburgh, Scotland; 2 NHS Education for Scotland, Edinburgh, Scotland, UK; 3 Scottish Centre for Simulation and Human Factors, NHS Forth Valley, Larbert, Scotland; 4 NHS Education for Scotland, Glasgow, Scotland; 5 Centre for Medical Education, School of Medicine, University of Dundee, Dundee, Scotland

**Keywords:** Professional identity, Narrative inquiry, Positioning theory, Workplace learning

## Abstract

Professional identity formation is central to developing resilient, ethical, and practice-ready healthcare professionals. While well-studied in medicine, professional identity formation in pharmacy remains underexplored. Recognising some of the unique challenges of the pharmacy profession, there is a need for empirically grounded studies that examine how pharmacy learners actively construct professional identities in real-world contexts. This nuanced perspective also adds depth and contextual relevance to the wider discourse on professional identity formation. This study examines how trainee pharmacists construct professional identities through narratives of challenging workplace experiences. Using Bamberg’s positioning analysis, we conducted a narrative inquiry into 13 micro-narratives derived from semi-structured interviews with trainee pharmacists in Scotland. Each narrative was analysed through three levels of positioning: self within the story, self in relation to the audience, and self in relation to broader cultural master narratives. We identified five core identity positions: *the transforming practitioner* (creative, value-adding problem-solver), *the shield-bearer* (protector of patient safety in the face of structural risk), *the quiet subversive* (agent of resistance), *the compassionate witness* (emotionally attuned and morally engaged observer), and *the shadow contributor* (invisible yet operationally essential team member). The findings highlight the emotional, relational, and narrative work underpinning identity formation. They call for educational practices that legitimise uncertainty, acknowledge and support emotional labour, and openly discuss the dissonance and complexity inherent in becoming a professional. Recognising and engaging with the identities learners inhabit, and indeed those that they resist, can foster more inclusive, authentic, and supportive environments for professional growth.

**Supplementary Information:**

The online version contains supplementary material available at 10.1007/s10459-025-10480-1.

## Introduction

Encompassing the multi-faceted and evolving sense of one’s professional self, professional identity formation (PIF) has emerged as a cornerstone of health professions education and scholarship. Contemporary discourse reflects the growing awareness that becoming a healthcare professional involves far more than acquiring clinical knowledge and skills; it is a process shaped by relationships, values, emotions, and the broader sociocultural context of practice (Cruess et al., 2019; Varpio et al., 2025). Over the past decade, scholars have increasingly questioned reductionist views of identity and have advanced relational, discursive, and critical perspectives that articulate the nuance and complexity of identity work. Professional identity is now widely understood as context-dependent and continuously negotiated (Varpio et al., 2025). It can be conceptualised as an internal state, whilst simultaneously also a performative, situated response to social expectations, institutional norms, and personal values (Sawatsky et al., 2023; Monrouxe & Rees, 2015; Liao et al., 2023).

This shift has given rise to a growing body of scholarship examining how identity is constructed through social connection and emotional labour. Newer models such as *PIF-as-functional-crafting* (Biesta, 2020; Varpio et al., 2025) emphasise the translation of identity into agentic action as an expression of one’s unique self within the profession. Such conceptual advances acknowledge that learners bring pre-existing identities and diverse cultural narratives to their training. Varpio et al. (2025) also highlight that the gap between what is expected by a profession, and what an individual actually does, can provide an opportunity for learners to undertake identity work that is personal and meaningful. In practice this might mean encouraging learners to identify gaps between the ways that they choose to behave and the behaviours that might be expected of their professional group within that context. In this way they might better understand their own carefully calculated acts of resistance or nonconformity (Vaa Stelling et al., 2023), thereby learning to situate and express themselves as unique individuals within the bounds of their profession.

However, despite this rich theoretical development, there is increasing disconnect between how PIF is conceptualised in contemporary scholarship and how it is enacted in educational settings. Formal curricula often default to abstract professionalism frameworks, checklist-based competencies, or unidimensional portrayals of professional ideals, leaving limited space for learners to explore ambiguity, vulnerability, or moral complexity in their identity work (Frost & Regehr, 2013). As Varpio et al. (2025) argue, persistent metaphors such as “fitting into a mould” continue to shape educational practice, even as research critiques their reductive implications. Meanwhile, early-career professionals across healthcare disciplines report increasing levels of burnout and identity dissonance, often linked to misalignment between institutional expectations and personal values (Vaa Stelling et al., 2023; Sawatsky et al., 2023). Whilst the majority of professional identity research within the health professions is undertaken in nursing or medicine, (Cornett et al., 2023), the pharmacy literature provides insights into some of the unique challenges within that profession.

Within pharmacy education, Noble et al.’s (2019) scoping review of 22 studies revealed that, while PIF is increasingly recognised as crucial in undergraduate pharmacy education, most reported curricular initiatives are fragmented, small-scale and delivered as discrete activities. They identified a lack of involvement of patients and interprofessional colleagues in identity-forming experiences, and highlighted the need for both empirical studies and longitudinal, integrated curricula that cultivate professional identity over time. Responding to these gaps, Janke et al. (2021) proposed a conceptual schema describing the core components of a pharmacist’s identity. They argued that a strong professional identity is essential to equipping graduates for contemporary pharmacy practice and drive transformation. They called for a step-change from ad-hoc interventions toward systematic embedding of identity-building touchpoints, supported by faculty development and institutional commitment. Together, this body of work emphasises that for PIF to be effective, it must be nurtured across the educational and practice continuum through authentic, relational experiences.

The findings of Noble et al. (2019) and Janke et al. (2021) resonate with recent conceptualisations within the wider health professions education literature, asserting that an essential ingredient of PIF is enabling students and trainees to actively inhabit, explore and negotiate their emerging professional selves. However, identity-forming experiences embedded in real-world practice settings remain poorly understood. Quinn et al. (2020), in a grounded theory exploration of early preregistration placements, found that students progressed through stages of reflection, attribute selection, professional socialisation and role perception, explicitly noted that the mechanisms by which experiential learning in practice contributes to identity formation are underexplored. Similarly, Kennie-Kaulbach et al. (2023) examined reflective writing from a community pharmacy rotation, emphasising the need to better understand how such workplace engagements shape evolving identities. Likewise, Kellar et al. (2023) identified the importance of experiential context as a core component of identity formation. As such, there is a need for empirically grounded studies that examine how pharmacy learners actively construct professional identities in real-world contexts.

This study addresses the gap by exploring trainee pharmacists’ workplace narratives, providing nuanced insights into how trainees discursively navigate their identities in practice. Research that explores the active, situated identity work that trainee pharmacists undertake in early practice contributes to wider discussions in health professions education by illustrating the identity positions learners inhabit during formative professional experiences. By linking these findings to contemporary theory, we aim to inform the development of educational practices that are both theoretically sound and practically responsive to the experiences of those in training within the health professions. Enhanced understanding offers educators, supervisors and policymakers targeted opportunities to intervene more effectively during early health professions training, thereby facilitating smoother transitions into practice, improving role clarity, enhancing professional confidence, and potentially mitigating early-career identity crises and burnout.

### Theoretical approach

The term ‘professional identity’ can mean a variety of different things, depending on the theoretical approach adopted. This study is situated within the social constructivist paradigm, meaning that identity is viewed primarily as a social phenomenon, influenced by culture, language and context. Through this lens, individuals construct multiple identities during interaction, continuously influenced by society’s culture, context and history (Monrouxe & Rees, 2015; Burr, 2015). Positioning theory (Bamberg, 2020) explores how individuals construct identities through discourse by adopting and assigning roles within interactions. It focuses on how people position themselves and others in relation to power, responsibility, and social norms, revealing how identity is dynamically negotiated through storytelling and everyday talk. Importantly, positioning theory draws distinction between roles and position – roles are relatively static and formalised, but positions, i.e. how individuals enact their roles, are dynamic, contextually-dependant and relational.

### Research question

We aimed to explore how workplace experiences, specifically the various positions that trainee pharmacists narrate for themselves, influence their construction of professional identity. This study therefore addressed the following questions:

1. How do trainee pharmacists position themselves within narratives of their workplace experiences?
2. How do the positions narrated by trainee pharmacists enhance understanding of their professional identity construction?

## Methods

### Study overview

In this study, we utilised a discursive, narrative approach to elicit stories about the daily work of trainee pharmacists. Narrative inquiry describes qualitative research methods whereby stories form the unit of analysis (Riessman, 2008). Identities reside in the stories we tell, in which we construct specific versions of ourselves and others. Positioning analysis (Bamberg, 2020) prioritises the specific positions that narrators construct within micro-narratives, taking context and the influence of ‘macro-discourses’ (master narratives; prevalent ways of representing people, groups or concepts) into account. Interviews designed to elicit micro-narratives of experiences were analysed using positioning analysis to understand how trainee pharmacists construct their identities through storytelling about their work.

### Ethics

Ethical approval for this study was granted by the NHS Education for Scotland Research Ethics Committee (approval numbers NES/Res/13/20/Ph and NES/Res/30/22/Pharm). Study participants were volunteers and received information about the study before consenting. All participants completed a written consent form and were free to leave the study at any time, without providing a reason. This study was conducted in accordance with the Declaration of Helsinki, which sets internationally recognised ethical principles to ensure that the rights, safety, and well-being of research participants take precedence over the interests of science and society.

### Context

Our study was conducted in Scotland. In Scotland, like the rest of the United Kingdom, aspiring pharmacists first complete a General Pharmaceutical Council accredited qualification (Master of Pharmacy; MPharm), after which they complete their foundation training year. During this foundation training year, trainee pharmacists undertake supervised practical experience in clinical settings such as community pharmacies, hospitals, or general practice. The year is designed to consolidate the knowledge and skills acquired during the MPharm degree, preparing trainee pharmacists for independent practice. Unlike other healthcare professionals, trainee pharmacists operate in a role traditionally associated with the supply of a tangible product, the filled prescription, rather than principally delivering an intangible service. This orientation can affect how the public and other healthcare providers perceive them, potentially seeing pharmacists more as dispensers than clinicians. Indeed, studies show that pharmacists are often viewed by physicians as ‘dispensers’ (Salim, 2016), and that the public frequently perceives pharmacists primarily as suppliers of medication rather than healthcare advisors (Hindi, 2018).

### Participant recruitment

Recruitment was facilitated by NHS Education for Scotland (NES), the body responsible for the training of postgraduate pharmacists in Scotland. We recruited trainee pharmacists attending a national education programme to the study on a voluntary basis. The programme consisted of immersive simulation-based scenarios and was delivered exclusively to trainee pharmacists at around the mid-point of their foundation training year. Author SMS was the primary organiser pf the program and was present during delivery of all simulation sessions. We sent an email invitation to all participating trainee pharmacist (185 out of a total of 219 trainee pharmacists across Scotland), and we provided those who responded to the initial email invitation with further information relating to the purpose of the study and content of the interview. Recruitment ceased when the sample size was deemed to have sufficient information power to address the research questions, based on the aims of the research, the specificity of the sample, the theoretical grounding of the study, the quality of the interview data, and the method of analysis (Malterud et al., 2016).

### Data collection

Between February and March 2025, author VT interviewed trainee pharmacists using an interview guide designed collaboratively by all authors to elicit narratives of workplace experiences occurring during the foundation training year. Given the wide geographical distribution of participants across Scotland and the significant travel distances this entailed, interviews were conducted online to facilitate inclusion and reduce logistical barriers. VT conducted narrative interviews, asking participants to share memorable or recent experiences. Specifically, participants were asked to share a story of a time they felt that they embodied the role of a pharmacist and, in contrast, a time they felt they didn’t belong in the role. Prompt questions such as ‘Can you give me more detail?’ and ‘How did that experience make you feel?’ were used to explore their thoughts and feelings relating to their experiences. The full interview guide is included as Appendix 1. Previously unknown to all participants, VT introduced herself as a ‘researcher’ with the aim of encouraging uninhibited discussion by de-emphasising any perceived power imbalance (Limerick et al., 1996). SMS and FS were not involved in the interview process as they were known to participants by virtue of their roles within pharmacy training. Interviews were audio-recorded and transcribed verbatim using MS Teams, cross checked by VT with the audio file, and anonymised.

### Data analysis

To increase familiarity with the data and identify discrete narratives, VT listened to all recordings, noting the start and end of each micro-narrative (hereafter referred to as ‘narratives’). A narrative was defined as a story describing a sequence of events linked by time, location and the specific characters involved (Riessman, 2008). Each narrative was analysed using the Bamberg et al.’s three levels of positioning analysis to delineate discursive construction of participant identities across narratives (Bamberg, 2004; Bamberg et al., 2011). Level 1 examines how the narrator positions themselves in relation to other characters within the story, specifically in relation to the pre-defined dimensions of sameness, agency and change during the story (Bamberg, 2020). Level 2 explores how the narrator positions themselves in relation to the audience (in this case the researcher), and their choice of goal-orientated conversational techniques. Finally, level 3 utilises the understanding of how the narrator positions themselves relative to other characters and the audience to analyse who the narrator is beyond the context of the story; how they position themselves within master narratives or macro-discourses. As the analysis proceeded, descriptions of the positions were presented by VT to SMS, FS and SES for discussion, debate, refinement and naming suggestions. In these team discussions, particular emphasis was placed on ensuring that the context, perspectives and norms of the pharmacy profession were taken into consideration throughout all stages of the study.

Once the positions constructed by trainee pharmacists were better understood, they could provide insights into professional identity construction. If an individual positions themselves in a similar way across multiple story-telling episodes, they construct an identity that endures beyond the stories themselves (Deppermann, 2015). Bamberg’s positioning analysis can therefore be utilised not only to explore how identities are constructed during storytelling, but also to provide insights into persistent elements of trainee pharmacists’ professional identities, constructed across a range of narratives by multiple individuals. This part of the analysis therefore addressed the second research question through identification of connections and cross-cutting themes in relation to the positions that had been discursively constructed in response to the first research question. This was done collectively by the research team, by grouping positions that seemed to emphasise similar aspects of professional identity, with deep discussion and debate relating to the breadth and meaning of each of the five core identities. In accordance with the Standards for Reporting Qualitative Research, trustworthiness was enhanced through prolonged and deep engagement with the data, searching for negative or contradictory narratives, maintaining an audit trail of analysis decisions, and engaging in frequent reflexive discussions (O’Brien et al., 2014).

### Reflexivity

Our research team consisted of researchers from diverse backgrounds, with different preconceptions and experiences relating to the research topic. SMS and FS are academic pharmacists with education leadership roles and significant experience of trainee pharmacists’ workplace roles, relationships and identity development. SES is a general practitioner with a strong qualitative research background, who has researched with VT (a hospital-based general physician) for over 15 years, together researching and publishing on various aspects of professional identity (Smith et al., 2024; Kerins et al., 2023a, b). The team have co-designed and undertaken a series of studies exploring aspects of trainee pharmacist professional development, drawing on each other’s strengths and experiences whilst comfortable to challenge each other’s preconceptions and engrained professional stereotypes (such as the risk averse nature of pharmacists and over-confidence of doctors). Reflexivity was enhanced through a cyclical process of reflective diarising and team meetings which included discussion of our preconceptions and expectations in relation to the research questions. The reflexivity process was particularly important within this study to ensure that the previous physician-centric identity research undertaken by VT and SES did not unduly influence analysis and interpretation within a pharmacy context.

## Results

Participants included 10 trainee pharmacists: nine who identified as female, and one who identified as male (from an overall population of trainee pharmacists with 80% identifying as female). Seven trainees were completing community-based programmes, two were on hospital-based programmes and one was undertaking a split community-hospital programme with experience of both settings. They were aged between 22 and 31 years old and were all at around the mid-point of their foundation training year. Interviews with each of the 10 trainee pharmacist participants lasted between 25 and 43 min.

Within the 10 interviews, we identified 23 discrete narratives, 13 of which we selected for deeper analysis. The 13 narratives were chosen to represent a range of practice contexts (hospital, community, primary care), offer clear identity navigation dilemmas, and contain rich emotional and professional reflections. Within the 13 selected narratives, eight were stories of embodying the role of a pharmacist and five were stories of times when they felt they did not belong in the role.

### The positions constructed by trainee pharmacists

Using Bamberg’s three-level positioning framework, each narrative was analysed in terms of how the trainee pharmacist positioned themselves within the story (level 1), in relation to the audience (level 2), and against broader professional or societal discourses (level 3). This process illuminated the ways in which identity was navigated through dilemmas of agency, sameness, and continuity (or change). Across the 13 selected narratives, a range of distinct identity positions were constructed, such as ‘*custodian’*, ‘*apprentice’* and ‘*protector’*. Table 1 details a summary of each of the 13 selected narratives, along with a summary of the relevant master narratives (main themes within the narrative) and self-positioning, culminating in the positions constructed within each narrative detailed in bold text in the right-hand column of the table. These positions capture the emotional, relational, and moral complexity of trainee pharmacists’ experiences and laid the foundation for the cross-narrative synthesis that followed.

**Table 1:** The positions constructed by trainee pharmacists through self-positioning against relevant professional and societal discourses (TP = trainee pharmacist code; N = distinct narrative code)

ID	Narrative summary	Master narratives	Self-positioning	Position and analytical notes
TP6, N12 Com	Reflects on increased autonomy in consultations and vaccine delivery. Describes shift from seeking supervision to feeling like a pharmacist, with emotional honesty around imposter syndrome and learning through responsibility	Safety, responsibility, experiential learning, emotional professionalism. Pushes back against rigid academic ideals	Maturing, reflective professional navigating fear and gaining confidence. Embraces imperfection and growth	**Pilgrim**: Captures the transitional nature of trainee identity, moving through doubt, reflecting deeply, taking on responsibility, and becoming agentic. An example of clinical, emotional, and moral growth
TP5, N10 Com	Discovers a dispensing error, investigates the audit trail, advises a colleague, and manages the reporting process. Reflects on how previously they would have relied on a qualified pharmacist but now acted independently	Clinical safety, error transparency, and pharmacist leadership	Claims authority and responsibility. Positions self as capable of leading and guiding others	**Custodian**: Shows transition from dependent learner to proactive, responsible team member, takes ownership. Embodies values of vigilance, leadership, and moral clarity
TP5, N11 Com	Faced with a diagnostic uncertainty involving a baby’s rash. Expresses discomfort, defers to pharmacist, and reflects on lack of clinical knowledge and confidence and worries about future responsibility and independence	Professional competence, patient safety, clinical responsibility, and preparedness for future roles	Overwhelmed learner, not yet ready to shoulder full pharmacist responsibilities	**Apprentice**: Identity tension; trainee is caught between rising expectations and internal doubts. Strong emotional insight into the weight of clinical responsibility and the fragility of self-confidence
TP4, N7 Hosp	Independently leads a discharge scenario, identifies necessary amendments, liaises with doctors, dispensers, community pharmacies, and the GP, and ensures safe patient transfer. Reflects pride and confidence in acting without supervision	Patient safety, leadership, communication, interprofessional collaboration, and pharmacist accountability	Independent decision-maker, patient advocate, and systems navigator.	**Coordinator**: Trainee reflects with clarity and pride on leading a complex clinical interaction, evidencing confidence, responsibility, and fluency in professional collaboration
TP4, N8 Hosp	Questioned by a doctor who expresses doubt about their ability to handle discharges. Trainee internalises this, leading to a lingering sense of inadequacy	Interprofessional respect and capability; supporting trainee autonomy	Less-than-legitimate pharmacist; undermined and stuck in an in-between state	**Outsider**: Account of how fleeting professional doubt or external scepticism can deeply impact self-perception. Highlights emotional labour of positioning oneself within medical hierarchies early in training
TP2, N4 Paeds hosp	Raises concern about a paediatric paracetamol dose, only to be dismissed and made to feel wrong. Reflects on the experience, its emotional impact, and how observing poor practice can be a form of learning	Clinical safety, ethical integrity, and moral responsibility. Questions cultural norms of silence or indifference	Ethical observer learning to define good practice through contrast and reflection	**Resister**: Narrative of moral clarity undermined by local culture. The trainee begins to define their professional identity in quiet but resolute resistance to complacency, while remaining quiet
TP3, N5 Com	Manages a challenging consultation alone, calmly defuses a patient’s distress, communicates medical reasoning, contacts the GP, and secures appropriate treatment. Reflects pride and emotional resilience	Communication, patient advocate, and gatekeeper	Resilient, autonomous, and clinically trustworthy; affirms readiness for role	**Problem-solver**: Highlights emotional regulation as professional strength. Trainee pharmacist presented as a steady, relational presence in patient-centred care
TP3, N6 Com	The trainee describes conducting a medication review independently, only for the pharmacist to redo it without acknowledging their input. This leads to feelings of invisibility and loss of confidence	Professional legitimacy, hidden labour, and resilience through rejection	Unrecognised contributor learning to endure and self-affirm	**Overlooked**: A subtle, emotionally resonant narrative of invisible labour. Demonstrates how being overlooked shapes identity through quiet, ongoing struggle to be seen
TP1, N1 Com	Assisting at the pharmacy counter, the trainee notices a prescribing issue, intervenes to prevent harm, and reassures the patient. Reflects on this as a meaningful first step into the pharmacist role	Patient safety, advocacy, professional accountability, and early leadership	Stepping into pharmacist identity by taking initiative and caring for vulnerable others	**Protector**: An example of early professional identity formation. A small act of initiative and care becomes a marker of belonging, with emotional sincerity and moral clarity driving self-positioning
TP1, N2 Com	Recounts an early mistake involving inappropriate antibiotic advice over the phone. Reflects deeply on feelings of guilt, lessons learned, and struggle to accept imperfection as part of professional life	Perfectionist ideals in pharmacy; fallibility, reflection and growth through error	Human learner; learning to hold that tension	**Self-critic**: A narrative that illustrates the identity work of reconciling perfectionism with human fallibility - a common but rarely articulated facet of early professional formation
TP10, N21 Com	Interaction with a homeless patient collecting methadone. They feel deep empathy and reflect on the patient’s vulnerability, the pharmacist’s social role, and their own discomfort and humanity	Patient-centred care, social justice, compassion in practice	Humanised practitioner, negotiating discomfort and emotional exposure	**Listener**: Shows the pharmacist as an empathic presence. Highlights emotional connection and moral humility as central to professional identity
TP8, N17 Com	Observes a pharmacist interacting dismissively with a patient and struggles with the internal conflict of knowing it was wrong but not speaking up. Reflects quietly on discomfort and the desire to do better in future	Silent complicity in hierarchical structures; speaking up	Passive witness hoping to become active advocate; moral identity in progress	**Observer**: Captures the tension of witnessing but not intervening, a critical identity moment. It highlights how silence is an act of positioning in early professional development
TP9, N18 Com	Receives an unexpected disclosure of sexual trauma from a patient. They navigate the moment with empathy, professionalism, and self-awareness, but later feel emotionally overwhelmed and unsure how to process it	Trauma-informed care, professionalism, and emotional labour	Strong but affected -emotionally capable but human	**Bearer**: Highlights emotional support as core to pharmacy identity. It offers insight into the invisible labour of absorbing patient distress, and how this shapes self-understanding

The practice setting relevant to each narrative follows the trainee pharmacist and narrative codes (Com = community practice; Hosp = hospital-based practice; Paeds hosp = paediatric hospital-based practice) GP = general practitioner

### Using the positions to enhance understanding of PIF

Following analysis of 13 selected narratives from trainee pharmacists using Bamberg’s three-level positioning analysis, we constructed five core identities through a process of cross-narrative synthesis and interpretation. These identities incorporated positions constructed by multiple individuals in a range of contexts, cutting across the distinction between narratives that described belonging and those that articulated not belonging. Each identity reflects a persistent way in which trainees discursively navigated and constructed what it means to be a pharmacist. The identities are not static roles, but symbolic positions negotiated through narratives of multiple participants to manage emotional, relational, and professional tensions. The five identities are described below, with examples from the data. The paraphrased examples use the words and expressions of individual participants but have been edited for brevity and to enhance clarity.

1. **Transforming practitioner: From doubt to competence**

This identity incorporates the positions of ‘pilgrim’ (TP6, N12), ‘apprentice’ (TP5, N11), ‘self-critic’ (TP1, N2) and ‘overlooked’ (TP3, N6). Trainees constructed this identity as a gradual process of internal transformation. They described moments of uncertainty, vulnerability, and imposterism, which gave way to growing confidence. These narratives centred on the emotional and experiential work of ‘becoming’. They constructed professional identity as the product of transforming emotional raw material through processes of trial and error, uncertainty and incremental realisation.

**Paraphrased example (summarised using phrases from TP6, N12)**

*“At first*,* I would panic when left alone with a patient. I wasn’t sure I could make decisions without checking everything. But over time*,* especially when no one was around to ask*,* I realised I did know what to do. I wasn’t just a student anymore - I was actually doing it. That realisation hit me after a few consultations in a row went well. I left thinking: maybe I am a pharmacist.”*

2. **The shield-bearer: Defending safety**

This identity was constructed through narratives of assertive action, leadership, and accountability. It amalgamates the positions of ‘custodian’ (TP5, N10), ‘coordinator’ (TP4, N7), ‘protector’ (TP1, N1) and ‘problem-solver’ (TP3, N5). Integral to these positions were trainees’ self-perceptions as salient to safe care and system integrity, often stepping up before being formally authorised to do so. These trainee pharmacists framed their identity as a proactively protective one, stepping into clinical and moral responsibility ahead of explicit invitation or formal recognition.

**Paraphrased example (TP4, N7)**

*“I led the whole discharge process - picked up the dosing issue*,* made the changes*,* liaised with the doctor and the dispensary*,* even found a new community pharmacy. No one told me to do it*,* I just knew it had to be done. When the patient’s daughter thanked me*,* it felt like I’d done something that mattered.”*

3. **Quiet subversive: Resisting wrongness**

The positions ‘resister’ (TP2, N4) and ‘observer’ (TP8, N17) revealed that some trainees constructed their identities in opposition to perceived poor practice or unsafe culture. Silenced or dismissed, these individuals identified themselves as morally aware and critically reflective, even when unable to act decisively. Their resistance was sometimes enacted, but sometimes internal. These trainees represent quiet subversives, defining their professional identity through resistance to what they refuse to become, in quiet defiance of unsafe norms and in commitment to integrity over professional compliance. This identity was complicated at times by hierarchical marginalisation whereby the predominance of doctors could, at times, limit their capacity to resist unsafe cultures.

**Paraphrased example (TP2, N4)**

*“I knew the dose was too high. I raised it*,* gently*,* but the pharmacist brushed it off. It made me feel like I should’ve kept quiet. Still*,* I know I was right. I’ve learned from watching what not to do - and I won’t ignore things just because others do.”*

4. **Compassionate witness: Practising humanity**

This identity was constructed through narratives of emotional labour by incorporating the positions of ‘bearer’ (TP9, N18) and ‘listener’ (TP10, N21), along with crossover with ‘self-critic’ (TP1, N2). These trainees identified as emotionally attuned practitioners, deeply affected by patient interactions. Whilst practicing emotional containment in the moment, their professional identities were entwined their ability to hold space for others, absorb emotion, thereby facilitating relational depth and moral presence.

**Paraphrased example (TP9, N18)**

*“The patient told me about their trauma. I wasn’t expecting it - I just listened*,* offered space. I handled it okay on the outside*,* but afterwards I couldn’t stop thinking about it. No one teaches you what to do with those feelings. It made me realise being a pharmacist isn’t just clinical*,* it’s emotional.”*

5. **The shadow contributor: Present but overlooked**

This core identity was marked by marginalisation and the experience of being undervalued. It coalesces from the position of ‘outsider’ (TP4, N8) crossing over with ‘overlooked’ (TP3, N6) and ‘observer’ (TP8, N17). Trainees identified as present, engaged, and often competent, yet repeatedly overlooked or excluded from meaningful participation. They contributed knowledge or effort, but without recognition or validation. Trainees constructed this identity through experiences of marginalisation and exclusion. Such experiences were further complicated by the unique product-oriented identity of pharmacists, embedded in public and interprofessional perceptions, which created additional challenges for trainees seeking recognition as service-providing clinicians.

**Paraphrased example (TP4, N8)**

*“The doctor asked if I was the pharmacist. I said I was a trainee - they just nodded and walked away. After that*,* I felt like I didn’t belong on the ward. Even when I spoke up*,* it felt like no one was really listening.”*

These five identities, while analytically distinct, may overlap and co-exist within the same individual. They are best envisaged as a pentahedron, five facets of identity that co-exist and inter-relate, with each face more or less visible depending on the orientation in space, in this context determined by a myriad of personal, social and professional factors. Together the identities offer a complex, but no doubt incomplete, account of professional identity construction as narrated by trainee pharmacists: a contested, dynamic, emotional, and relational negotiation of who they are and who they are becoming.

## Discussion

This study explored how trainee pharmacists discursively construct their professional identities through micro-narratives of workplace experience, analysed using Bamberg’s three-level positioning framework. Across 13 selected narratives, five core identity positions were constructed: *the transforming practitioner*, *the shield-bearer*, *the quiet subversive*, *the compassionate witness*, and *the shadow contributor*. These identities represent negotiated responses to emotional, relational, and moral tensions, echoing contemporary conceptualisation of PIF within the health professions as a dynamic, socially situated and deeply personal process (Cruess et al., 2019; Jarvis-Selinger et al., 2012; Varpio et al., 2025). The identities are fluid and continuously shaped by the distinct tensions of the pharmacy profession, such as occupying a healthcare role that is uniquely tied to the supply of a product – the filled prescription. This duality of product and service orientation informs how trainee pharmacists narrate their roles, negotiate relationships, and construct their professional identities (Kellar, 2020).

The identity of *the transforming practitioner* (transforming doubt into confidence) aligns with broader educational discourse that positions uncertainty and vulnerability as core drivers of identity development, suggesting that moments of dissonance often catalyse the internal assimilation of professional values (Kerins et al., 2023b). In our study, identity was constructed through a process of realising one’s competence through emotional labour, moving through doubt and uncertainty. The conceptualisation of PIF as active, ongoing ‘work’, shaped by emotional dissonance, builds on recent research that concludes (in the context of medicine residents) how heavy, and at times overwhelming, such identity struggles can be (Sawatsky et al., 2024) Whilst situated in a social context, identity work is often deeply isolating, and the burden of self-doubt is frequently carried alone. This resonates with Noble et al. (2019) who found that students reported limited opportunities to engage with others to explore their professional identities. Yet such work remains of critical importance, as it is through these difficult experiences that professional identity develops and transforms (Sawatsky et al., 2018). These identity-critical experiences, situated within the unique context of the pharmacy profession, therefore need to be recognised and supported, rather than buffered or avoided.

Similarly, the identity of *the compassionate witness* resonates with Varpio et al.’s (2025) emphasis on how relations with others, particularly in contexts and situations where there is no single ‘right’ course of action, are foundational to finding one’s unique place within a profession. Narratives in our study featured trainee pharmacists sharing patients’ distress and navigating emotional spillover in ways rarely captured by formal curricula or guided by professional standards. These findings build on work that has utilised the tripartite model of meaning (coherence, purpose, value) to explore how PIF contributes to wellbeing and fosters resilience through meaning-making (Toubassi et al., 2023). Our work calls for greater attention to be paid to the association between PIF and emotional resilience, aspects of professional development that seem to interact in more intricate and synergic ways than has been previously recognised.

In this study, *the quiet subversive* identity draws attention to how trainee pharmacists experience, and at times resist, the implicit norms and power structures of the workplace. This identity reflects an active tension between institutional expectations and personal values, influenced by professional hierarchies and the unique pressure to prioritise dispensing speed over safety. Learners described moments of silencing, dismissal, or exclusion, responding without cynicism or disengagement, but with internalised critique and resolve. Our work reinforces the notion that identity is often forged through conflict and that learners construct who they are not only by emulating role models, but also by identifying and resisting what they refuse to become. Recent work exploring advocacy and resistance in medical education goes further, acknowledging that along with individual acts of silent resistance, there can be, at times, more overt demonstrations of tension between one’s own personal values and those aligned with the professional collective with which they identify as a member (Ellaway et al., 2025). Recognition of these deeply personal (and, at times, seemingly confrontational) forms of professional identity work can help educators, supervisors and institutions support learners to navigate the complexity of the clinical workplace critically and reflectively. Indeed, interprofessional education may be valuable in facilitating meaningful conversations relating to power dynamics and engrained professional hierarchies, enabling trainees to reflect on and possibly reconfigure them. Our previous work (Tallentire et al., 2022) has suggested that such conversations are likely to be important in fostering collaborative attitudes and changing workplace-based behaviours. In addition, creating space for narrative sharing and reflexivity may be key to supporting identity formation that is robust in the face of moral challenge and is attuned to traditional medical hierarchies.

### Implications for practice

This work set out to illustrate the identity positions inhabited by learners during formative professional experiences, as a stepping stone towards bridging the gap between contemporary identity theory and the development of educational practice. It is through work such as this that the conceptual advancements evident in the scholarly literature can be enacted within training programmes to facilitate transitions, mitigate burnout and nurture colleagues who are at ease with the dynamic, multi-faceted nature of their professional identities.

The findings suggest that PIF in trainee pharmacists may be shaped to a significant extent by emotional labour, moral tensions and resistance to cultural norms. Building on the work of Kellar et al. (2023) illustrating the plurality of pharmacist identity and the salience of a healthcare provider identity, this study suggests that educators should consider how workplace learning environments support the narrative, emotional, and ethical dimensions of becoming a professional. Practical strategies might include structured reflective opportunities, supported by faculty development in narrative elicitation and exploration (Mann et al., 2009). Recognising identity positions such as *the transforming practitioner* or *the shadow contributor* invites a more compassionate and inclusive approach to supervision that validates learners’ emotional experiences, explores moments of dissonance, and supports the development of resilient, relational identities. However, this work echoes previous calls for such individualistic strategies to be adopted alongside those emphasising socialisation or relational recognition. The identities of *the shield-bearer* and *the shadow contributor* foreground integration, participation, and teamworking in an interprofessional context as central to identity development. It therefore follows that strategies facilitating inclusion and visibility in clinical teams, such as interprofessional simulation or other modalities that promote reflecting with others in a safe space, may help to foster positive identity formation in trainee pharmacists (Mount et al., 2022; Tallentire & Smith, 2024).

### Strengths and limitations

This study explored PIF within a national cohort of trainee pharmacists, using narratives grounded in their individual workplace experiences. Whilst the five core identities are firmly rooted within the pharmacy context, the issues that they highlight in relation to the complex, multi-faceted and dynamic phenomenon of PIF may resonate more widely within health professions education. However, the study was limited by some important constraints. Firstly, the sample size of 10 trainee pharmacists is relatively small and although the data were rich and plentiful, it may be that a larger cohort of participants might have led to additional conceptual insights. The 13 selected narratives were chosen for their depth of reflection and to represent a range of practice contexts. However, a different set of narratives is likely to have yielded different insights and during the selection process, certain themes that resonated with the researchers’ interests or experiences are likely to have been prioritised. Finally, the trainee pharmacists may have tried to paint themselves in a positive light to ‘save face’ with the researcher, and it is likely that there are also some negative influences on PIF that are not fully captured here.

## Conclusion

This study advances contemporary understanding of PIF by exploring the nuanced ways trainee pharmacists construct and navigate their professional selves through workplace narratives. In the underexplored context of pharmacy training, we used positioning theory to identify five core identities that reflect learners’ emotional, moral, and relational negotiations in response to real-world dilemmas. These identities highlight that PIF is not merely a linear or cognitive process, but a dynamic, context-sensitive interplay between individual agency, emotional labour and social expectation. Many of the identity tensions described, such as moral dissonance and emotional spillover, are rarely acknowledged in formal curricula. Our findings suggest that educational strategies must extend to support the narrative and emotional dimensions of identity work. This includes fostering reflective spaces, validating uncertainty, and embracing the messiness of becoming, in both personal and relational ways. In these ways, educators can facilitate more humane, resilient, and inclusive transitions into professional life.

## Supplementary Information

Below is the link to the electronic supplementary material.

Supplementary Material 1

## Data Availability

I can provide examples of all levels of data analysis but they are not included with the submission (other than a summary table as Table 1) due to the volume of qualitative data involved.
