# Supplementary material for: Stories of who we are: exploring trainee pharmacists’ professional identity constructions through workplace narratives
Source: Adv Health Sci Educ Theory Pract. 2025 Oct 3;31(3):909–25. doi: 10.1007/s10459-025-10480-1 (PMC13233982; doi:10.1007/s10459-025-10480-1)
Supplement: Supplementary file 1 — Supplementary Material 1 [file 10459_2025_10480_MOESM1_ESM.docx]

**Appendix 1: Interview guide**

*Pre-interview checks:*

*Informed consent process*

*Confirm region, programme type and progress through Foundation training year*

I am keen to hear stories of your work as a trainee pharmacist, relating to recent memorable experiences. Please provide as much detail as you can remember, whilst trying to avoid the names of patients or colleagues.

1. Can you start by telling me the story of a time when you really *felt* like a pharmacist, when you really embodied that role?

Prompt questions: Can you say more about that?

How did that experience make you feel?

Can you provide more detail?

What do you feel now about that experience?

1. In contrast, can you tell me about a time when you *didn’t feel* that you belonged in the role of a pharmacist, when you didn’t embody the role?

Prompt questions: Can you say more about that?

How did that experience make you feel?

Can you provide more detail?

What do you feel now about that experience?

1. Do you have any questions for me or the research team about this project?

*Thanks, provide contact details and any other supports required.*
